# Supplementary material for: Are carbon emissions trading and green financial instruments synergistic? -Comprehensive quantitative research based on content analysis
Source: PLoS One. 2024 Mar 7;19(3):e0298601. doi: 10.1371/journal.pone.0298601 (PMC10919601; doi:10.1371/journal.pone.0298601)
Supplement: S1 Data — (ZIP) [file pone.0298601.s001.zip › Data/地级市Z_F工作报告.zip!/┴╔─■/▒╛╧¬╩╨/└┤╫╘╣1⁄2╓┌║┼í╛┬φ┐╦╩2╛▌═°í┐_2016.docx]

各位代表：

　　我代表本溪市人民政府向大会报告工作，请予审议，并请市政协委员和各位列席同志提出意见和建议。

　　一、“十二五”时期及2015年工作回顾

　　过去的五年，在省委、省政府和市委的坚强领导下，全市各族人民开拓进取，攻坚克难，基本完成了“十二五”规划确定的主要目标任务。

　　——经济综合实力再上新台阶。“十二五”时期，全市生产总值累计完成5618亿元，年均增长8.5%；各项主要指标稳步增长，为全面建成小康社会奠定了坚实基础。

　　——产业结构调整迈出新步伐。以“三都”建设为抓手调结构、促发展，绿色钢都建设深入推进，中国药都建设成效显著，中国枫叶之都品牌形象逐步确立，服务业发展提速升级，智慧城市建设起步良好。三次产业比例由2010年末的5.0:62.3:32.7调整到5.7:51.8:42.5。

　　——城乡面貌发生新变化。成功获得国家园林城市称号，创建国家卫生城市通过省验收，成为首批省级环保模范城市。主城区基础设施和城市功能进一步完善；太子河新城启动建设，城市向北拓展框架基本拉开；沈本新城建设空间达25平方公里、集聚人口超10万；两县县城规模均达到20平方公里。公路、铁路路网密度进一步增加。生态环境质量持续改善，全力实施“青山、碧水、蓝天”工程，关停关闭小高炉等重点污染源300余个，全市森林覆盖率达到76%。

　　——改革开放实现新突破。完成两轮行政审批制度改革和三轮简政放权，取消和下放行政职权841项，减少行政审批事项386项。落实商事登记制度改革，五年累计新登记各类市场主体46383户。对外开放进一步扩大，五年累计出口125.9亿美元，成功引进一批国内外知名企业。沈本一体化持续推进，实现沈本通信等一体化。高新区获批成为国家级高新区。

　　——人民生活有了新改善。连续五年为民办实事，有效实施了棚户区改造、“暖房子”工程、教育、医疗、就业、社保等一批群众关心关注的民生工程。城镇和农村常住居民人均可支配收入年均增速分别达到11.7%和12.8%。平安本溪建设持续深化，社会和谐稳定局面继续巩固。

　　“十二五”时期，全市经济社会实现质量和效益协调统一的可持续发展，“三都五城”新本溪建设取得了实质性进展。“十二五”的发展实践证明，只要我们坚定不移地将这张好的蓝图绘到底，本溪一定能够在振兴发展的道路上走得更好、走得更远！

　　各位代表，刚刚过去的2015年是面临发展形势最严峻、经济下行压力最大的一年。一年来，我们认真学习贯彻习近平总书记系列重要讲话精神，结合本溪实际，落实“4+2”着力要求，坚持“4+3”驱动共同发力，培育壮大“6+2”新增长点，迎难而上，主动作为，推动了全市经济社会持续稳定发展。预计2015年，全市生产总值完成1160亿元，公共财政预算收入实现53.5亿元，固定资产投资600亿元，社会消费品零售总额360亿元，出口创汇24.5亿美元，城镇、农村常住居民人均可支配收入分别为27530元和12550元。

　　过去一年，主要做了以下五方面工作：

　　（一）全力以赴稳增长，经济保持平稳运行

　　“项目年”活动取得较好成效。重点推进300个亿元以上项目，已竣工88个，国电投本溪热电、辽中环高速本溪段、田桓铁路、观音阁水库输水工程等一批新建、续建重大项目进展顺利。桓仁抽水蓄能电站项目预可研通过国家审查。思山岭铁矿开发获省正式核准。大力推进PPP模式，首批在省平台公示项目16个，计划总投资161亿元。集中开展“百日空间会战”，李家山改造等58个空间会战项目彻底销号。

　　抢抓政策机遇效果明显。全力争取上级政策、项目、资金支持，共争取上级专项转移支付资金59.5亿元，有力地支持了南芬独立工矿区和溪湖老工业区搬迁改造等项目建设。先后申报四批国家专项建设基金项目，其中列入国开行、农发行拟签约项目23个，资本金注入8.5亿元，拉动投资170亿元。同时，与国开行、农发行深度合作，筹措城建交通及太子河新城建设资金22.6亿元。积极争取地债资金、地下综合管廊项目资金14.2亿元，用于城市基础设施建设和社会事业发展。歪头山独立工矿区改造搬迁纳入国家支持范围正在报批中。

　　服务工业企业平稳运行。市委、市政府成立八个稳增长推进组，帮助协调解决企业生产、运行、资金等各类问题500余项。9大工业产业集群平稳发展，生物医药和钢材深加工产业集群呈现亮点，葡萄酒、包装印刷等特色产业集群集聚效应明显提升。落实国务院资源税调整方案等减负政策，减轻企业税费负担5.6亿元、电费2.3亿元。

　　支持本钢集团稳定生产。认真落实市政府与本钢集团签订的战略合作框架协议，着力解决本钢历史遗留问题及制约发展的瓶颈问题，全力推进本钢冷轧高强钢等项目建设。本钢积极应对钢铁行业困境，采取强力措施保持生产运营基本稳定，为全市稳增长做出了重要贡献。发挥本钢带动作用，绿色钢都6个产业基地和3个平台建设持续推进，金桥焊材等重点项目产能逐步释放。

　　编制完成“十三五”规划。按照贯彻落实十八届五中全会精神和省委、市委全会提出的《建议》要求，以项目为核心，通过专题调研、专项推进、专家论证等方式，超前谋划经济社会发展蓝图，编制完成全市“十三五”规划纲要及大健康产业等31个专项规划。筛选866个项目重点推进，为“十三五”持续健康发展提供支撑。

　　（二）创新思路促改革，发展活力不断释放

　　重点领域各项改革实现突破。编制完成“4+3”驱动、“6+2”新增长点实施方案并进入实施阶段。完成三轮简政放权，取消调整行政职权892项，公布权力清单和责任清单，行政审批实现网上办理。实施“三证合一、一照一码”登记制度改革，“三证合一”登记494件。完成县区市场监管体制“三合一”改革。财税体制改革稳步推进。“两税”通过联合办税深度融合。全面启动国家城市公立医院改革试点。开展了农村土地确权登记。东北林业产权交易中心、本溪不动产登记中心正式挂牌。启动国资国企改革，制定出台具体实施意见和监管办法。大力推进价格改革，全面实行城市居民用水、燃气阶梯价格制度，启动了供暖收费标准调整工作。

　　投融资体制改革取得进展。筹备和组建城建、旅游、水务等行业性控股公司，启动水洞景区旅游体制改革试点。探索并实践政府对专项资金投入竞争性领域的项目采用基金形式注入，通过市场化运作实现保值增值。总规模5亿元的生物医药创业投资基金、健康产业投资基金已投入运营，筹备组建的东北智慧城市等产业（创业）投资基金工作正在全力推进。

　　对外开放扎实推进。坚持“走出去、请进来”，全市共洽谈项目475个，签约项目105个，新开工项目74个。协调跟进对接央企、沪企、知名民企“辽宁行”活动项目76个，其中上海医药北方药业、天士力健康产业园等15个项目正式落户。建立了海关、出入境检验检疫和外经贸“三位一体”工作服务机制。本溪市保税仓库正式投入使用，为发展跨境电子商务奠定基础。

　　（三）坚定不移调结构，转型升级步伐加快

　　中国药都“三化”进展顺利。规模化方面，全域药都建设初具规模。上海绿谷等18户企业新开工或复工，药联制药、恒康制药等20户重点医药企业销售收入同比增长35%。以北京同仁堂、龙宝参茸等龙头企业为支撑，以“北药”、健康食品、保健品为主的大健康产业起步良好。国际化方面，日本卫材制药落户药都，韩国大熊制药完成设备安装，晟麦、大石等企业出口持续增长。城市化方面，沈阳药科大学主校区完成50万平方米建设任务，中国医科大学盛京医院本溪基地二期工程试运营，辽宁中医药大学本溪校区和辽宁医药职业学院正在进行主体施工。沈本新城高铁站、客运枢纽站等建成投入运营。

　　枫叶之都建设亮点纷呈。本溪水洞风景区升级为国家5A级景区。先后发布“枫红指数”、主要景区负氧离子数据。全力推进智慧旅游平台建设。打好温泉项目建设攻坚战，益东温泉小镇花溪沐会馆一、二期相继投入运营，天著希尔斯温泉酒店、温泉谷项目主体完工，益东温泉中心开工建设。多渠道强化城市形象推广和旅游产品宣传，成功举办桓仁野山参暨国际冰酒博览会、国际极限越野挑战赛等系列主题活动。以关门山、枫林谷等重点景区和“十一”黄金周为代表的旅游市场持续火爆，全年旅游业总收入增长15.2%。

　　服务业保持良好发展态势。金融业发展活跃，税收达到10亿元，其中金融机构贷款余额突破1000亿元，同比增长14.4%。物流业发展势头良好。电子商务加快发展，高新区获评“国家电子商务示范基地”，两县获批全国电子商务进农村综合示范县。成功举办2015中国医药50人论坛、北方环境论坛暨李家山生态高峰研讨会、铁刹山“道教·中国”养生文化论坛等高端活动。商贸流通业市场布局加快，桓仁东北参茸城正式营业，本溪市农产品交易中心项目签约落地。

　　农业实现快速发展。服务“三农”水平增强，基本农田得到有效保护，全市粮食作物和蔬菜播种面积稳定在80万亩以上。市级以上农业产业化龙头企业达到119户。家庭农场、合作社等新型农业经营主体达到884个。本溪县、桓仁县获国家休闲农业与乡村旅游示范县。农产品质量安全监管体系实现市县乡全覆盖。农业标准化水平稳步提升，“三品”认证企业35家、面积97万亩。“一县一业”健康发展，初步形成“企业+基地+农户”的产业化发展模式。本溪县刺五加、桓仁县葡萄酒两个农产品加工集聚区初具规模。完成千万亩经济林工程3.7万亩。完善小型农村水利灌溉工程，新建农村饮水安全工程34处。

　　智慧城市建设取得阶段性成果。信息惠民等5个国家试点项目建设进展顺利。我市获得中欧智慧城市创新奖、腾讯智慧城市奖、政府服务奖等殊荣。成功举办信息惠民国家试点城市经验交流会。由我市制定的全国第一个省级智慧城市地方标准已发布。市民融合服务平台完善升级，市民网提供多项政务服务。全国第一张加载居民健康、金融、交通等服务内容的市民卡已制发60万张。扁平化决策系统接入全市31个部门的业务系统和数据。云数据中心一期、医疗卫生、教育信息等平台建成使用。

　　（四）积极稳妥防风险，保重点、保稳定取得实效

　　积极应对政府债务风险。构建了“借用还”一体化的融资偿债体系，初步建立投融资归口管理、政府债务风险管控体系，争取地方政府置换债券49.6亿元，全部偿还了2015年到期政府债务。

　　积极应对社会保险金支付风险。通过强化养老保险基金征缴、争取上级专项转移支付资金等方式，多措并举，确保了企业离退休人员养老金的按时足额发放。采取政府补助等方式，确保工伤、医保基金支付。

　　积极应对财政支付风险。严格执行新《预算法》，加强财政预算执行和管理，强化收入征管和非税收入监管。优化支出结构，压缩一般性支出。积极筹措资金，实现保工资、保运转、保民生目标。

　　积极应对社会稳定风险。毫不松懈地抓好安全生产、食品药品监管、技术监督等工作，杜绝重特大事故发生。深入推进领导干部接访下访等工作，有效化解一批突出信访问题和矛盾隐患。扎实抓好平安本溪建设，创新完善立体化社会治安防控体系建设，严厉打击各类违法犯罪活动，加大公共安全管理力度，维护了全市社会大局的持续稳定。

　　（五）不遗余力惠民生，各项社会事业稳步发展

　　就业和社会保障保持稳定。全力推动“大众创业、万众创新”，完成个转企1046户，新增创业2409人，城镇实名制就业4.88万人，城镇登记失业率3.96%，控制在省市目标以内。完成省定社保扩面任务。统筹提高多项社保待遇，其中企业退休人员养老金月人均增加205元、达到2101元；城乡低保标准分别提高7%和10%。建立了临时救助制度。实施精准扶贫，减少国标贫困人口4105人。

　　生态环境保护力度加大。我市获评全国绿色发展优秀城市，“创模”和“城市环境总体规划试点”得到环保部肯定。在省内率先开展生态文明示范市建设工作。桓仁县被列入全国第一批生态保护与建设示范区名单，本溪县被列入全国第二批生态文明先行示范区建设名单。实施“青山、碧水、蓝天”工程，全市整体环境质量得到改善，全年环境空气质量二级以上良好天数达75%以上。

　　棚户区改造任务全面完成。争取国开行棚改贷款28.1亿元，全面完成省政府下达的新增保障性安居工程12221套任务。创新货币化安置方式，实施“政府团购”，全市棚改货币化安置率达90%，消化存量商品房近40万平方米。牛心台、卧龙、彩北安置区3000余户棚改居民搬进新居。

　　城市管理不断加强。完成卫峪街等66条街巷路整治工程。实施“暖房子”工程338.8万平方米，惠及居民6.7万户。物业管理业主大会筹备、组建率达90%以上。编制完成停车系统规划，新建公共停车场21处，新增停车泊位1315个。开通延伸6条公交线路。完成平山路等6条主干路综合整治及北地长江广场周边地区环境整治。

　　部分城市建设难题有效解决。全力攻坚城市建设中的难点和遗留问题。平山甲楼是全市最大的棚户区，改造项目涉及住宅楼88栋、居民4976户，目前动迁工作已经结束；停工14年的胜利路三角地改造项目重新启动建设；历时5年的小堡畜牧场和历时8年的地工路改造动迁遗留问题彻底销号。克服资金、动迁、施工等困难，沈丹客专、北地跨线桥实现全线通车。

　　宜居乡村建设取得阶段性进展。6个宜居示范乡镇基本达到“五美”标准，39个宜居示范村实施了“六化”建设。完善垃圾处理、畜牧生态养殖等基础设施，改造村内道路851公里，村屯绿化119个，改造农村危房686户。

　　各项社会事业同步发展。教育改革积极推进，南芬区、溪湖区（含高新区）、桓仁县通过省县域义务教育均衡发展审核性评估。制定现代职业教育实施意见和建设规划。公共文化服务体系建设步伐加快，深入实施“十惠”文化民生工程，“社区书屋”实现全覆盖。两县公立医院改革平稳有序开展。成功承办CBA联赛等全国赛事，举办系列群众体育活动，掀起全民健身新热潮。外事、侨务、对台、民族、宗教、人防、档案、史志、气象等工作取得积极进展，妇女儿童、残疾人等事业得到新发展。

　　去年承诺的十大类民生工程19件实事，除1件未完成外，其余18件实事全部完成。

　　在2015年工作中，我们自觉接受人大的法律监督、工作监督和政协的民主监督，把办理好人大代表建议和政协提案作为政府工作的切入点和着力点，作为政府自身建设的强大动力，人大代表建议和政协提案问题解决率达71.6%。加快建设法治政府，顺利通过国家“六五”普法检查验收。

　　在2015年工作中，我们把开展“三严三实”专题教育作为政府自身建设的重要抓手，以只争朝夕、马上就办的工作作风狠抓落实，切实提高了政府工作效能。坚决落实党风廉政建设责任制，履行“一岗双责”，进一步强化了行政监察和审计监督。

　　各位代表，2015年及“十二五”发展所取得的成就，归功于省委、省政府和市委的正确领导，得益于市人大、市政协的监督支持，凝聚着全市人民的智慧和汗水。在此，我代表市政府，向全市人民、社会各界，向支持和参与本溪发展建设的朋友们，表示衷心的感谢和崇高的敬意！

　　当前，本溪正处于“滚石上山、爬坡过坎”的关键阶段。过去的一年，虽然我们付出了比以往更多的努力，但受内外部环境、主客观因素影响，总体发展成果尚未达到预期，经济指标增速回落，个别重点项目没有如期完成。我们清醒地认识到，本溪发展还面临不少困难和问题。从长远看，本溪仍处于老工业基地城市和产业转型升级的关键时期，解决经济结构调整、县区经济发展不平衡、基础设施不完善、社会事业发展滞后、高素质人才短缺、资源环境约束趋紧等矛盾还需付出长期艰苦努力。从近期看，经济社会发展也出现一些新情况、新问题：一是经济下行压力逐步加大。大宗产品行业面临产能过剩局面,项目引进竞争加剧，消费热点不多，出口出现负增长。二是实体经济面临不少困难。大部分冶金采选等资源型企业停产半停产，民营经济和小微企业发展瓶颈亟待突破。三是财政收支矛盾加大。受宏观经济下行和政策性减收因素影响，财税收入下降幅度较大，而社会保障、偿还债务等刚性支出不断增加。四是民生改善任务依然繁重。人民群众在城镇住房、市区交通、环境质量等方面的需求还未得到满足，就业、教育、医疗等公共服务领域仍需加大工作力度。五是政府职能转变和作风建设长效机制有待巩固完善。部分政府工作人员没有主动适应经济发展新常态，担当作为不够、服务意识不强、群众观念不牢、作风建设不到位、行政问责不到位。对这些问题，我们一定要高度重视，认真加以解决。

　　二、“十三五”时期主要目标和总体要求

　　各位代表，“十三五”是全面建成小康社会的决胜阶段，是推进东北老工业基地全面振兴的关键时期。我们要积极抢抓新一轮东北振兴机遇，坚定发展信心，明确发展目标，落实“五个必须”要求，加快建设“三都五城”新本溪，确保如期全面建成小康社会。

　　“十三五”时期发展的主要目标是：经济综合实力再上新台阶，主要经济指标增幅保持全省前列，自主创新能力逐步增强，发展质量和效益进一步提升，重点领域和关键环节改革全面深化，对外开放形成新格局，产业结构调整取得长足进展，城乡面貌显著变化，生态环境持续提升和改善，总体实现基本公共服务均等化，社会大局和谐稳定。到2020年，全市生产总值、城镇和农村常住居民人均可支配收入三项指标均实现比2010年翻一番。全市30584名贫困人口全部实现脱贫，完成全面建成小康社会目标，本溪人民生活更加富裕、更加幸福。

　　“十三五”时期发展的总体要求是：必须适应经济发展新常态。始终坚持发展第一要务，坚持变中求新、变中求进、变中突破，以提高经济发展质量和效益为中心，加大供给侧结构性改革力度，努力提升发展活力、内生动力和整体竞争力。必须树立五大发展理念。切实把创新、协调、绿色、开放、共享发展理念落实到振兴发展全过程。必须落实“4+2”着力要求。突出深化改革治本之策，抓住结构优化关键之本，把握创新驱动决胜之要，筑牢民生改善稳定之基，在推进城乡区域协调发展和改进干部作风上彰显新作为。必须激活“4+3”驱动。同步实施创新、改革、市场、开放和服务、主体、人才驱动，实现发展动力转换，打造本溪经济升级版。必须培育“6+2”新增长点。在传统产业转型升级、新兴产业发展、现代服务业发展、推进新型城镇化、转变农业发展方式、发展民营经济和金融、信息化上培育新增长点，打造经济发展新支撑、新引擎。

　　我们相信，经过未来五年的再努力、再创新、再奋斗，本溪全面建成小康社会目标一定会如期实现！

　　三、2016年工作任务

　　今年工作的总体思路是：以全面建成小康社会为总揽，以深入实施东北老工业基地全面振兴战略为先导，紧紧围绕“4+2”着力要求，坚持稳中求进工作总基调，聚焦“两个前列”目标，落实“两个双稳”任务，狠抓改革攻坚，突出创新创业，强化风险防控，加强民生保障，建设“三都五城”，努力实现“十三五”良好开局。

　　今年全市经济社会发展的主要预期目标是：生产总值增长4.5%左右，公共财政预算收入增长5%，固定资产投资（按新制度）增长6%，社会消费品零售总额增长8%，城镇和农村常住居民人均可支配收入与经济发展同步。

　　全年要重点做好六个方面工作：

　　（一）着力完善体制机制，推动改革开放取得新突破

　　以更大的智慧和勇气，深入推进改革开放，坚决破除体制机制障碍，为经济社会发展注入新动力、增添新活力、拓展新空间。

　　积极推进行政管理体制改革。全力抓好简政放权放管结合优化服务，积极推进“五个砍掉一批”，完善四个清单制度。继续深化“三证合一、一照一码”登记制度改革。稳妥推进事业单位分类改革。进一步做好清理规范行政审批中介服务工作。深化公共资源交易管理体制改革。按省统一部署完成公务用车制度改革。

　　深入推进财税体制改革。按照国家统一部署，适时实施各项税制改革。继续完善财政预算管理制度，加强政府债务管控，建立财政存量资金定期清理机制，完善权责发生制的政府财务报告制度，推进政府购买服务等政府采购制度改革。鼓励引导社会资本积极参与公共服务领域投资运营，拓宽公共设施建设融资渠道。

　　大力推进投融资体制改革。加快组建行业性控股公司并投入运营。积极推进设立产业（创业）投资基金，完善基金项目储备库，支持更多项目建设。加快推动生物医药、“互联网+”等3支创投基金和3支产业基金组建工作，支持基金投资的域外企业到本溪兴业。全力推进农信社改制，加快市商业银行深化改革。支持龙宝参茸等企业上市。

　　探索推进农村土地制度改革。落实农村土地集体所有权，稳定农户承包权，盘活土地经营权。全面开展农村土地承包经营权确权登记办证工作，推进农村土地确权登记53万亩。规范引导农村土地经营权有序流转。

　　深入推进林业综合改革。发挥我市作为全国林业改革综合实验示范区优势，组织实施国有林场改革。依托东北林业产权交易中心推进森林资源流转试点，加快林业产业化进程。以桓仁县为改革试点县，探索“三权分离”、以林抵押贷款工作，促进林业经济有序健康发展。

　　全力推进职业教育体制改革。坚持就业导向谋划发展职业教育，加强职业教育学校特色专业、品牌专业以及多种形式的实训基地建设，逐步分类推进中等职业教育免除学杂费，吸引和培养更多的高素质劳动者和技能人才。整合职业教育资源，推进产教融合、引企入校工作，探索组建适应本溪经济社会发展需要的职业教育集团。

　　稳妥推进国资国企改革。建立统一管理、分类监管的国有资产监管体系，实行监事派驻和国资稽查制度。对商业经营类国有企业按照“分类指导、一企一策”原则，采取整合重组、股权转让等多种途径，有序推进混合所有制改革。多方筹措资金，加大力度解决国企历史遗留问题。

　　大力发展民营经济。完善支持民营企业的政策措施，努力减轻企业负担，培育壮大一批重点民营企业和中小企业，增强民营经济对全市发展的贡献力。放宽民间资本进入条件，鼓励我市民营经济参与PPP项目建设、大健康产业、现代服务业等领域发展。

　　积极扩大对外开放。抢抓“一带一路”等战略机遇，加快推进我市产能输出和国际产能合作。全力扶持本钢出口，重点实施中小出口企业“二个一批”工程，形成新的出口增长点。抓好出口品牌和平台建设，努力扩大非钢产品出口份额。扎实开展招商引资活动，继续深化与央企、沪企和知名民企合作。

　　（二）着力推进结构调整，推动发展质量效益提升

　　促进供给侧和需求侧两端共同发力，推进结构性改革，着力培育壮大“新动能”，提升改造“旧动能”，形成接续财源，为经济保持中高速增长提供强大动力。

　　加快建设实力更稳固的绿色钢都。全力支持本钢落实省政府“一企一策”改革。继续服务本钢调整产品结构，重点推动本钢冷轧高强钢等项目尽快释放产能。推进高端绿色钢材、装备制造、精品铸件、钢材深加工、冶金综合利用、绿色矿山资源和新材料六大基地建设，深度挖掘“人参铁”品牌优势，突出发展精品铸造、精密部件制造，推动思山岭铁矿等项目建设，继续做优做强钢铁产业链。

　　加快建设内涵更丰富的中国药都。培育健康制造业，发展覆盖全生命周期的健康服务业，打造以大健康产业为内涵的全域药都。提升企业实力，强力推进高新区恒康制药、华润三药等96户存量企业达产增效，推进上药集团、上海绿谷等增量企业加快建设。促进校地合作，充分发挥沈阳药科大学等高校对药都建设的带动和辐射作用。继续完善高新区区域公交体系，加快城市功能建设步伐。抓紧编制沈本城际连接带建设规划，推动沈本一体化进程。充分发挥全市药材资源优势以及同仁堂、天士力等龙头企业带动作用，建设覆盖药材种植、中药制造、保健养生等独具本溪特色的健康产业集聚区，提升全域药都发展水平。

　　加快建设形象更鲜明的中国枫叶之都。以改革为引领，实施旅游“十百千万”总体布局，推动文化旅游、体育旅游、农业休闲观光旅游、森林旅游、健康养生旅游协调发展，培育特色鲜明的枫叶之都。丰富智慧旅游平台应用功能。完成本溪水洞整体改制。围绕打造温泉之城，加快重点温泉项目建设。积极发展沟域经济，建设星级农家乐示范区，培育一批采摘、现代农业、植物观赏等特色乡村旅游项目。着力引进旅游综合体，落实航空旅游、自驾营地、主题酒店等新业态项目。继续推出登山、骑行、露营等旅游系列产品。在央视黄金时段全年投放城市形象广告，加强旅游宣传促销。

　　全力攻坚项目建设升级年。全年考核推进340个亿元以上项目，重点实施108个工业、84个服务业、64个基础设施类项目，为经济结构优化、城镇化发展提供支撑。深入开展“百日空间会战”,破解空间要素等难题，加快项目开工建设。继续加大争政策、抢机遇力度，重点实施好溪湖老工业区和南芬独立工矿区搬迁改造项目，推进歪头山独立工矿区改造搬迁工程纳入国家支持范围。继续做好专项建设基金项目申报工作。加快国电投本溪热电、观音阁水库输水工程等重大项目建设，力争桓仁抽水蓄能电站项目前期工作取得突破。

　　全力攻坚工业提质增效。继续支持本钢创新发展，整体推进9大工业产业集群快速发展，存量企业实现提质增效，增量企业促进结构调整。落实国家、省《中国制造2025》部署，全面组织实施本溪“制造+智造”2025强市建设，重点推进实施智能化改造等5个方面、38个支撑项目和12个试点示范项目。坚持淘汰落后产能，加快传统产业升级。

　　全力攻坚服务业多元化发展。深入落实服务业发展四年行动计划，以发展服务业重点产业为抓手，努力补齐服务业短板。大力发展物流业。重点提升桥北、彩北物流园区建设水平，引进大型家居家装企业落户园区，推进本溪市农产品交易中心等项目建设。促进金融业发展。推动更多的金融机构入驻本溪，保持金融机构在本地贷款余额的增长势头。深化与国开行、农发行等金融机构合作，吸引更多资金注入项目建设。发行药都二期债。加快发展电子商务。依托本溪市保税仓库，大力发展跨境电商。搭建“枫乡本溪”和“绿色桓仁”商城，以电子商务进农村为发展注入活力。推动消费市场持续活跃。引入国内知名的大型商业综合体落户本溪，完善“夜经济”聚集区、特色街区建设。促进房地产平稳发展。用足用好国家推进保障性安居工程建设等政策，发挥好住房公积金作用，控制开发用地规模，积极消化房地产存量。

　　全力推进现代农业发展。全面实施“北药”发展规划，推进国家级（工厂化）中药材种苗繁育基地建设，启动订单药材种植模式，推动农产品精深加工，实现一、二、三产业深度融合。抓好“一县一业”提质增效。大力发展绿色生态农业，扶持“三品一标”认证和特色农产品品牌创建。加强耕地保护，搞好土地后备资源储备。推进农业融资平台建设，加快培育家庭农场等新型农业经营主体。探索“互联网+农业”发展模式。大力发展林下经济。加强流域、山洪沟治理及自然封育工作。加快畜牧业结构调整，形成标准化、规模化养殖，确保不发生区域性重大动物疫情和畜产品质量安全问题。

　　（三）着力鼓励创新创业，不断激发经济发展内生动力

　　坚持创新驱动引领，完善“产政学研资介”一体化创新体系，为加快转变经济发展方式提供支撑。

　　强化企业创新主体地位。发挥大型企业创新骨干作用，支持建设企业技术中心、工程（技术）研究中心、重点实验室，推进沈阳药科大学国家级制剂工程技术中心建设和申报，推进上海绿谷糖类药物省级重点实验室年内获批。加大科技型中小微企业培育力度，增强创新活力。建立高新技术企业培育库，重点扶持入库企业。

　　强化产学研创新成果转化。建立一批开放性公共科技服务平台，继续深化与沈阳药科大学、辽宁中医药大学、东北大学、辽宁科技学院等高校和科研机构的合作，搭建科研成果就地转化平台。加快组建产业技术创新战略联盟，推动产学研协同创新，年内组建高新区大型科学实验仪器共享联盟。

　　积极营造良好引智环境。进一步加强人才创新创业基地建设，出台有针对性的人才政策，创新引智模式，吸引更多的高素质人才、高水平团队来溪创业就业，将“大众创业、万众创新”各项政策落实到位，使“双创”成为经济发展的新引擎。

　　扎实推进智慧城市建设。巩固智慧城市建设全国领先地位，重点在惠民项目应用上实现新突破，完成智慧社区养老、教育信息等工程，打造本溪县“智慧满乡”、明山区“智慧体育”特色项目。在惠企上完成智慧产业项目建设，夯实国家“宽带中国”试点，以与国家工信部中国电子信息行业认证的战略合作为突破，在高新区建设认证与数据平台，拓宽中关村智慧创新平台，实现起点高、专业强的大数据智慧应用。

　　（四）着力保障和改善民生，让人民群众有更多获得感

　　民之所盼，政之所为。我们要按照保基本、兜底线、促公平、可持续的原则，努力保障和改善民生。

　　提高居民生活保障水平。继续落实援企稳岗政策，千方百计稳定就业。提高全市最低工资标准。开展“全民参保登记计划”，努力完成社保基金征缴任务，确保社保支出。建立统筹协调的社会救助体系，提高城乡最低生活保障标准。完善住房保障体系，合理有序增加公租房房源供给。推进居家养老市场化、社会化发展，加快养老服务设施项目建设。继续加大对残疾人等弱势群体的帮扶力度，全年发放残疾人辅助器具2000件，完成贫困残疾人家庭无障碍改造500户。

　　实施精准扶贫。按照国家“六个精准”扶贫要求，落实“五个一批”行动计划，推动72个驻村工作队有效帮扶，抓好贫困劳动力技能培训，一村一策、一户一策，推进和完善项目到户扶贫，充分发挥扶贫资金扶持作用，加强产业化扶贫基地建设，确保完成年度脱贫任务。

　　办好人民满意教育。继续构建教育强市。开展“义务教育均衡发展工作创建年”活动，确保各县区全部通过义务教育均衡发展省检。加快建设高新区“一城两区”教育集聚区，开工建设市高中民办国际分校，支持服务沈阳药科大学完成整体搬迁，推进辽宁医药职业学院加快搬迁、辽宁中医药大学本溪校区基本建成，为药都乃至本溪发展提供智力支持和人才支撑。

　　加快医疗卫生事业发展。继续推进城市和县级公立医院综合改革，实施高血压、糖尿病2个病种的分级诊疗，提高公立医院精细化管理水平。巩固加强乡村卫生服务体系建设，提升社区卫生服务能力，完善国家基本药物制度，保障患者根本利益。推进中国医科大学盛京医院本溪基地康复疗养中心正式运营。

　　推动文化体育产业发展。广泛开展文化惠民工程。提升文化软实力，建设书香社会，发挥文化精神引领作用。恢复建设市艺术宫。深入挖掘辽砚、剪纸、农民画等地域文化资源，推动特色文化产业加快发展。深化文化体制改革，鼓励社会资本进入演艺院线建设，壮大演艺产业。继续打造CBA、女乒、羽超等品牌赛事，促进体育产业与大健康等相关产业融合发展。推动全民健身，加强群众体育基础设施建设，提高全民健康素质。

　　推进平安本溪建设。强化社会治理创新，严厉打击各种犯罪，积极打造立体化社会治安防控网络新格局。全力抓好信访稳定、安全生产等工作。推进市食品检验检测中心、市质量技术检验检测中心建设，开展各类专项整治，确保食品药品安全。支持国防和军队建设，巩固全国双拥模范城成果。

　　各位代表，经过广泛征求社会各界意见建议，今年市政府将全力做好10项民生实事：一是实现1万贫困人口脱贫；二是完成棚户区改造9827户、农村危房改造1797户；三是实施“暖房子”工程300万平方米，同步实施住宅区综合整治工程9万平方米；四是打通育龙路至本桓线、千金路至丹霍线城市出口，实现太子河郑家大桥竣工通车；五是维修改造街巷路30条，改造人行道板20万平方米；六是全面启动主城区停车管理系统建设，新增停车泊位2000个；七是开工建设本宽线北大岭隧道及引线旅游环线工程，在旅游交通节点、旅游景区，新建和改造旅游公厕40处；八是主城区22所符合开放条件的中小学体育场馆全面向市民开放；九是建设市中心医院太子河新城医院和市失能半失能老人康复养护中心；十是推进智慧城市建设深度应用，制发100万张市民卡，实现市民就医、缴费、保险、交通、图书借阅等普惠。

　　（五）着力统筹推进城乡建设，促进区域协调发展

　　尊重城市发展规律，用改革思维加强城市规划建设管理，深化生态文明城市创建进程，推进新型城镇化，建设美丽本溪。

　　加强城市规划建设管理。严格执行城市建设规划，不断完善规划体系。全面加强城市建设，搞好城市绿化美化，加强城市公园、广场建设。完善城市基础设施，建设地下管廊，推动海绵城市建设步伐。推进工业余热利用工程，构建清洁、绿色供暖格局。推进垃圾焚烧发电项目建设。着力强化城市管理，抓好老旧小区改造，提升物业管理水平，分期分批拆除违章建筑。采取措施，有效解决“弃管小区”管理问题。采取有效措施治理城市拥堵。重点对水塔路、地工路等6条城市主干路环境进行综合整治，不断提升城市环境水平。

　　加快实现主城区向北拓展。以太子河城市风光带建设为载体，加快建设太子河新城，尽快完善新城基础设施和公共服务设施，拉开城市骨架、拓展城市空间、疏解城市人口。加快推动新溪湖·印象建设步伐。

　　全力构建“大交通”格局。围绕城市道路衔接贯通、城乡交通更加畅达，完善公路路网，提高公路等级，构建“大交通”格局。完成田桓铁路征地拆迁任务；推进辽中环高速本溪段、沈环线卧龙至三家子段改建工程建设，加快本桓高速、本宽高速前期工作；启动重建彩屯大桥，支持溪湖区改造建设鲍家两洞桥，新建、改造威宁大街、北光路等6条城市道路。

　　积极创建生态文明城市。启动编制《本溪市生态文明建设示范市创建规划》。继续开展“创模”、“创卫”、“创生态市”工作，大力实施“青山、碧水、蓝天、沃土”工程，重点开展低空污染整治、综合治霾，确保环境空气质量二级以上良好天数继续保持在75%以上。大力促进循环经济发展，保护和改善环境，实现可持续发展。倡导文明祭祀，推进移风易俗，实施市殡仪服务中心、殡仪馆搬迁改造工程。

　　扎实推进新型城镇化。加大规划引导力度，完善建设两县县城，培育建设一批功能健全和特色鲜明的小城镇，打造成为集聚产业、吸纳就业的有效载体。抓好宜居乡村建设，打造一批具有历史文化、田园风光、乡村旅游等特点的特色村。实施本桓公路两侧沿线整治提升工程。继续推进农村垃圾等环境治理，加强农村人畜饮水安全保障，努力提高农村基础设施和公共服务水平。

　　（六）着力加强政府自身建设，努力建设人民满意政府

　　实现2016年乃至“十三五”发展目标任务，政府肩负着重要责任。我们一定鼓足干事创业勇气，提升推动发展能力，进一步集聚正能量、释放精气神、全力促振兴。一要加强学习，坚定发展信心。主动适应经济发展新常态，完善政府系统党组（党委）学习制度，认真学习习近平总书记系列重要讲话精神，深化对十八届五中全会以及中央、省、市经济工作会议精神的理解，以更坚定的信心、更有效的举措抢抓机遇、推动发展。二要敢于担当，坚持勤政为民。强化为民服务宗旨意识，落实“两个前列”要求，完善干部目标绩效考核制度，提高政府效能，用敢于担当的勇气、善于担当的智慧和体现担当的成效，将国家、省和市委的决策部署落实到位。三要牢记使命，坚决依法履职。自觉接受人大的法律监督、工作监督和政协的民主监督，广泛接受社会各界和新闻舆论监督。启动“七五”普法依法治市工作，促进行政权力规范透明运行，切实提高依法行政能力。四要廉洁从政，坚守党性原则。抓好党风廉政建设责任制的落实，严格执行“一岗双责”。巩固“三严三实”专题教育成果，严格遵守政治纪律、组织纪律、廉洁纪律、群众纪律、工作纪律和生活纪律，认真贯彻落实《准则》和《条例》，营造风清气正的良好环境，形成干事创业的强大合力。

　　各位代表，抢抓东北新一轮振兴发展机遇，实现到2020年全面建成小康社会目标，是时代赋予我们的光荣使命和神圣职责。让我们在市委的坚强领导下，坚定信心，埋头苦干，为实现“十三五”良好开局，为谱写本溪经济社会发展新篇章而努力奋斗！
